# Supplementary material for: HIF-2α is indispensable for regulatory T cell function
Source: Nat Commun. 2020 Oct 6;11:5005. doi: 10.1038/s41467-020-18731-y (PMC7538433; doi:10.1038/s41467-020-18731-y)
Supplement: Supplementary file 1 — Supplementary information [file 41467_2020_18731_MOESM1_ESM.pdf]

Supplementary Information

Hypoxia-inducible factor 2α is indispensable for regulatory T cell function

Hsu T.-S. et al.

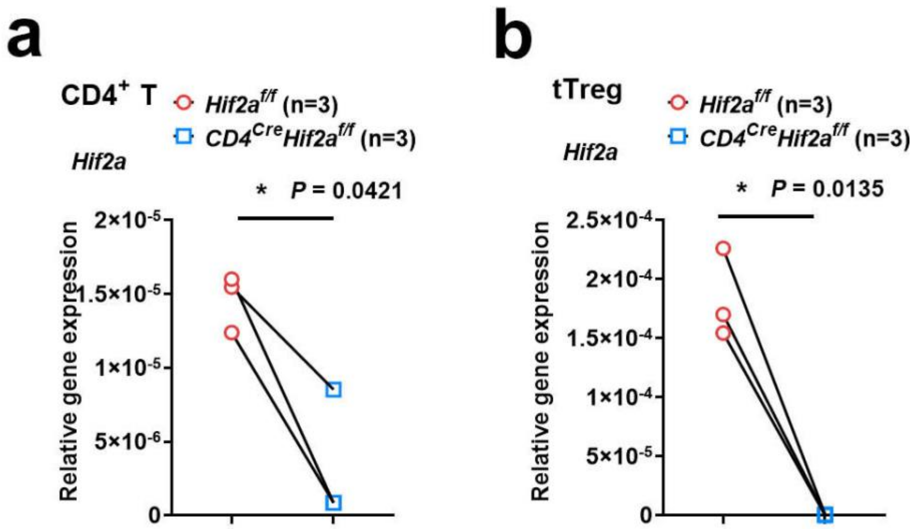

Supplementary Figure 1. Deletion of *Hif2a* from naïve T cells and tTreg cells. Naïve T cells (**a**) and tTregs (**b**) from *Hif2a$^{f/f}$* and *Cd4^{Cre}Hif2a$^{f/f}$* mice were sorted by FACSAria II SORP, and expression of *Hif2a* was determined by quantitative PCR. Samples were normalized to *Actin* expression. Red circle, *Hif2a$^{f/f}$*; blue square, *Cd4^{Cre}Hif2a$^{f/f}$*. n = 3 mice. Statistical analyses were performed using parametric, paired t-test, two-tailed. **P* = 0.0421 (a) and 0.0135 (b). Source data are provided as a Source Data file.

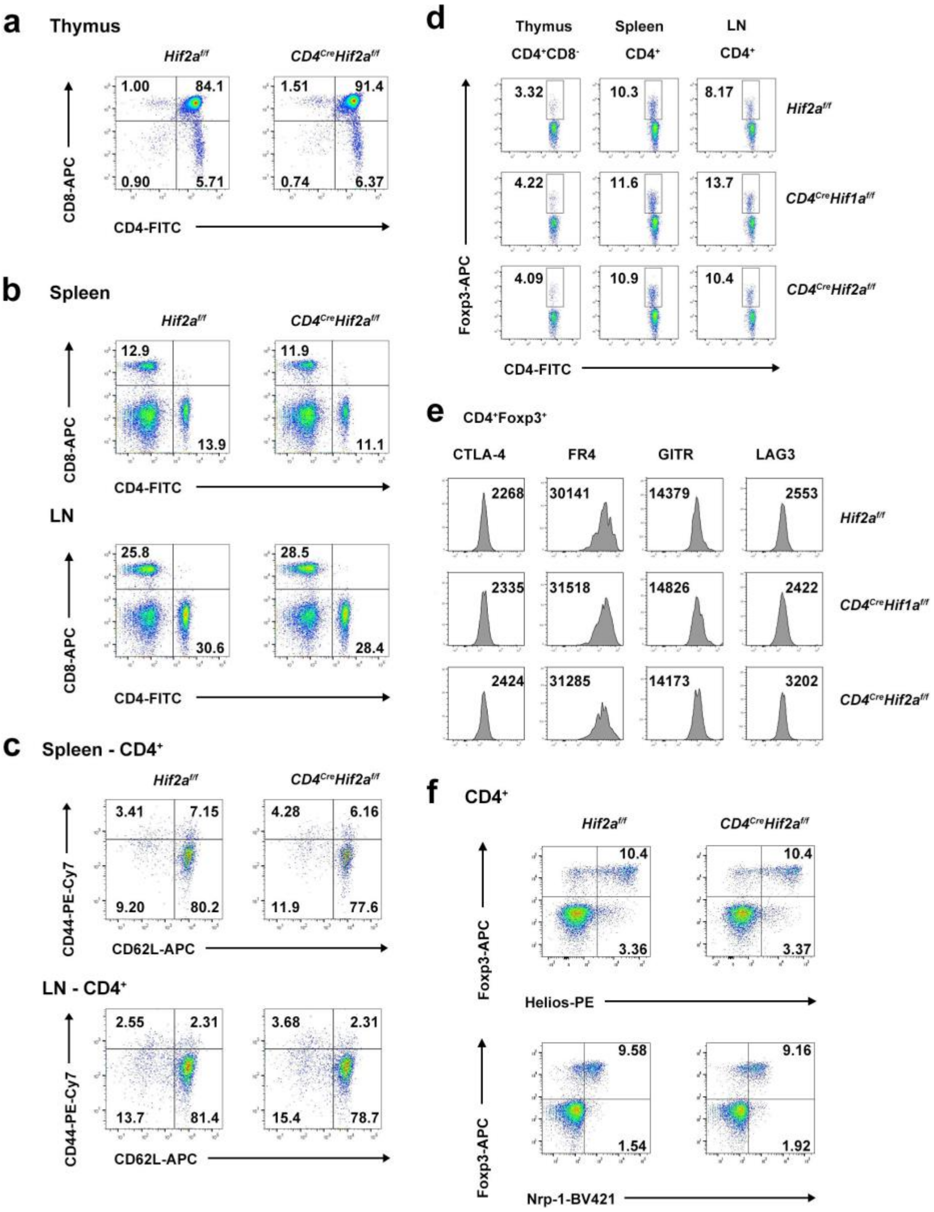

Supplementary Figure 2. Normal T cell development upon T cell-specific deletion of *Hif2a*. **(a, b)** Normal T cell development in *Cd4*$_{Cre}$*Hif2α*$_{f/f}$ mice. The distribution of CD4$_{+}$ T cells and CD8$_{+}$ T cells in thymus (**a**), spleen and lymph nodes (LN) (**b**) of WT (*Hif2α*$_{f/f}$) and *Cd4*$_{Cre}$*Hif2α*$_{f/f}$ mice was quantified. **(c)** Normal naïve and memory T cells in *Hif2α*$_{f/f}$ and *Cd4*$_{Cre}$*Hif2α*$_{f/f}$ mice. Splenic CD4$_{+}$ T cells from *Hif2α*$_{f/f}$ and *Cd4*$_{Cre}$*Hif2α*$_{f/f}$ mice were gated, and the CD44$_{hi}$CD62L$_{lo}$ and CD44$_{lo}$CD62L$_{hi}$ cell populations were determined. **(d)** Normal CD4$_{+}$Foxp3$_{+}$ cell population in *Cd4*$_{Cre}$*Hif1a*$_{f/f}$ and *Cd4*$_{Cre}$*Hif2a*$_{f/f}$ mice. CD4$_{+}$Foxp3$_{+}$ T cell populations in thymus, spleen, and peripheral lymph nodes of *Hif2a*$_{f/f}$, *Cd4*$_{Cre}$*Hif1a*$_{f/f}$, and *Cd4*$_{Cre}$*Hif2a*$_{f/f}$ mice were determined by flow cytometry. **(e)** Neither HIF-1α nor HIF-2α deficiency affects Treg phenotype. Expression of CTLA-4, FR4, GITR and LAG-3 in *Hif2a*$_{f/f}$, *Cd4*$_{Cre}$*Hif1a*$_{f/f}$, and *Cd4*$_{Cre}$*Hif2a*$_{f/f}$ CD4$_{+}$Foxp3$_{+}$ T cells was determined by flow cytometry. Number indicates mean fluorescence intensity. **(f)** Expression of Foxp3, Helios and Nrp1 in tTregs of *Cd4*$_{Cre}$*Hif2α*$_{f/f}$ mice is normal. CD4$_{+}$ T cells from *Hif2a*$_{f/f}$ and *Cd4*$_{Cre}$*Hif2α*$_{f/f}$ mice were analyzed for expression of Foxp3, Helios and Nrp1. Data (a-f) were confirmed in three independent sets of mice.

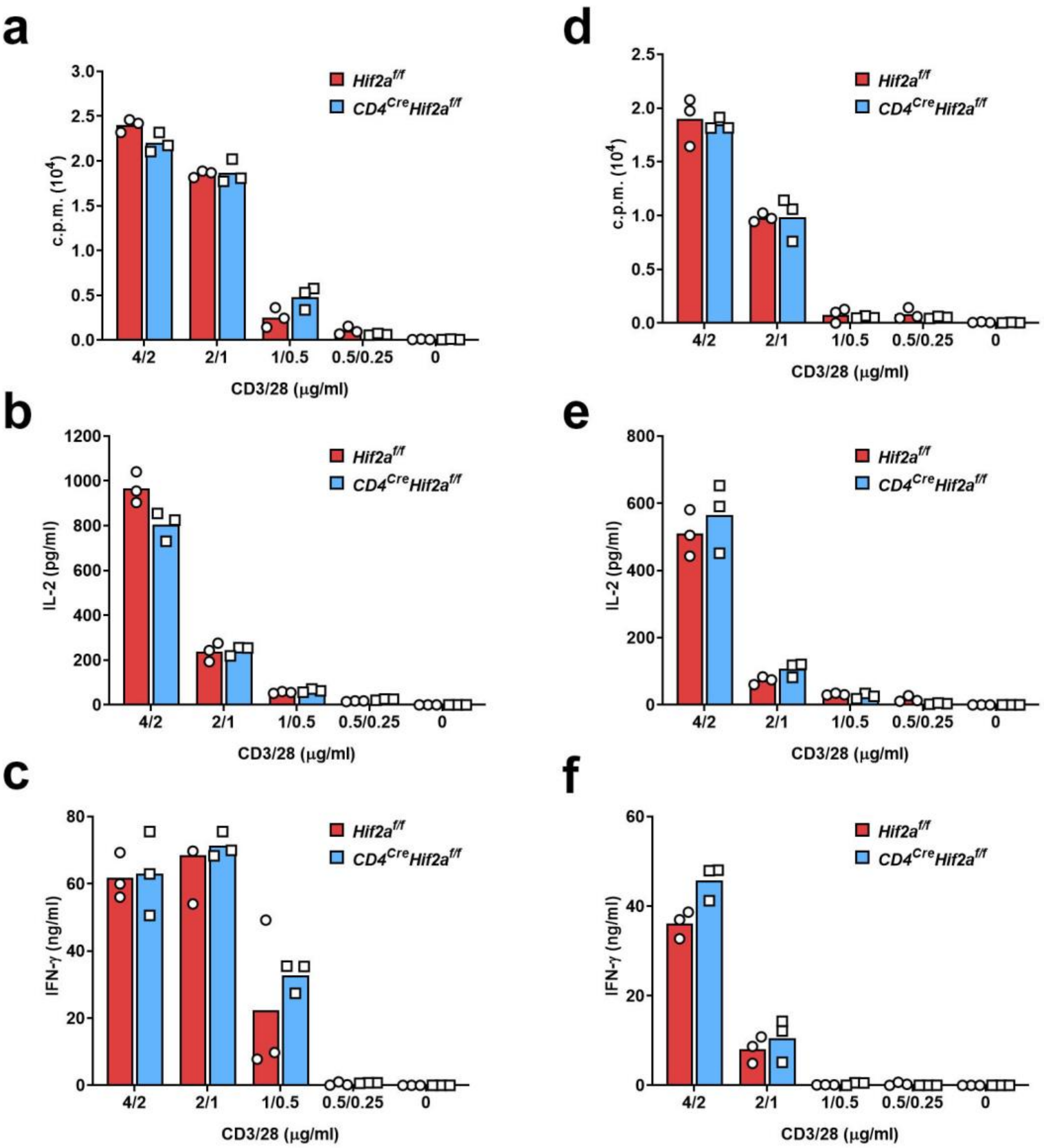

Supplementary Figure 3. Normal T cell activation in *Hif2a$^{-/-}$* *T cells*. T cells from spleens (**a-c**) and peripheral lymph nodes (**d-f**) of *Hif2a$^{f/f}$* and *Cd4^{Cre}Hif2a$^{f/f}$* mice were activated with different doses of anti-CD3/CD28. Culture supernatants were collected 40 hours after T cell activation, and production of IL-2 and IFN-γ in supernatants was detected by ELISA. T cell proliferation was measured by ^{3}H-thymidine incorporation 60 hours after T cell activation. c.p.m., counts per minute. Data are technical triplicates of a single experiment. Results have been reproduced in two independent experiments. Source data are provided as a Source Data file.

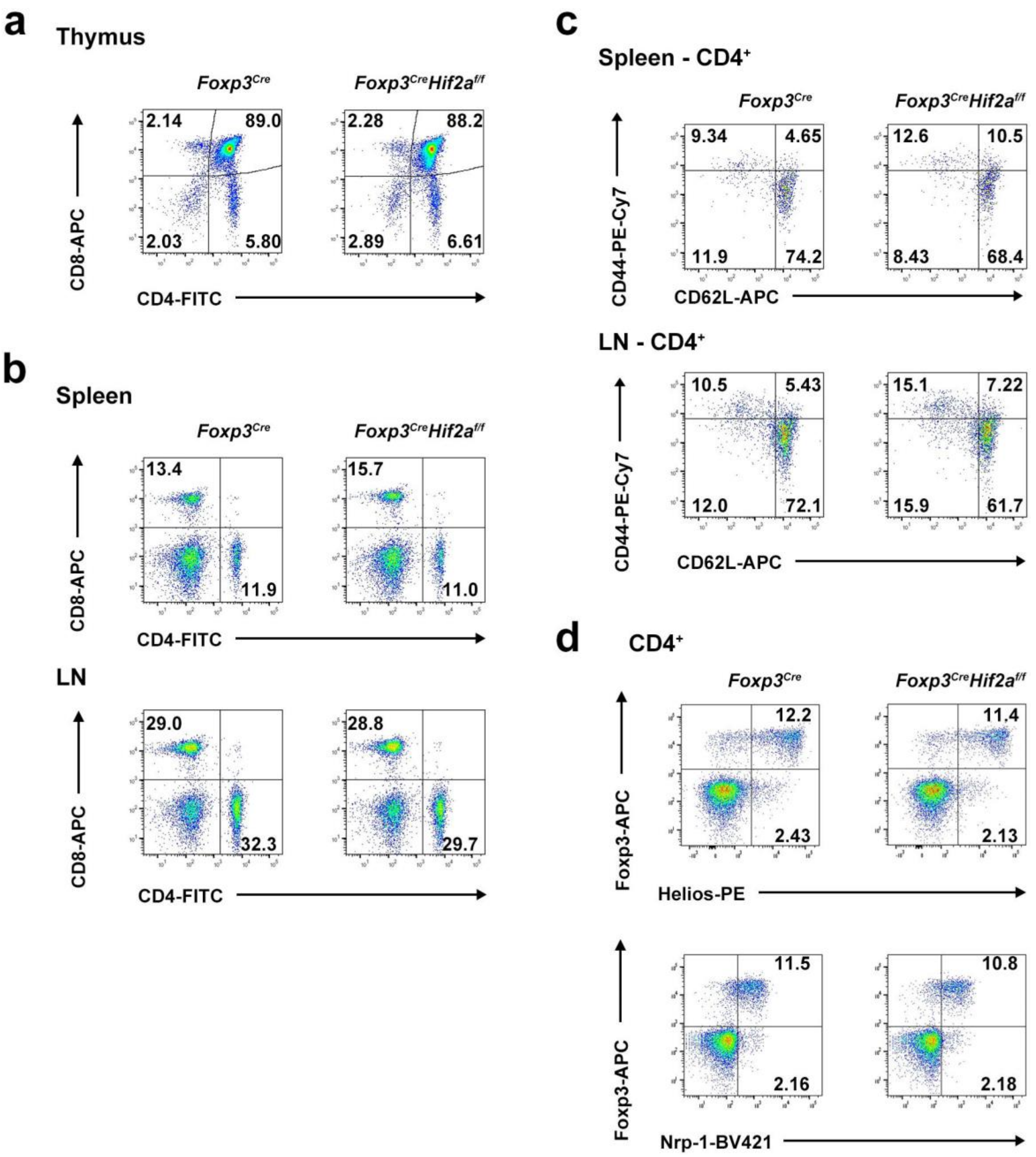

Supplementary Figure 4. Normal development of T cells upon Treg-specific deletion of *Hif2a*. **(a, b)** Normal T cell development in *Foxp3*^{Cre}*Hif2α*$^{f/f}$ mice. The populations of CD4^{+} T cells and CD8^{+} T cells in thymus (**a**), spleen and lymph nodes (LN) (**b**) of WT (*Foxp3*^{Cre}) and *Foxp3*^{Cre}*Hif2α*$^{f/f}$ mice were determined. **(c)** Normal naïve and memory cells in T cells from *Foxp3*^{Cre} and *Foxp3*^{Cre}*Hif2α*$^{f/f}$ mice. CD44^{hi}CD62L^{lo} and CD44^{lo}CD62L^{hi} cell populations in CD4^{+} T cells from spleen and lymph nodes (LN) of *Foxp3*^{Cre} and *Foxp3*^{Cre}*Hif2α*$^{f/f}$ mice were quantified. **(d)** Development of tTregs in *Foxp3*^{Cre}*Hif2α*$^{f/f}$ mice is normal. CD4^{+} T cells from *Foxp3*^{Cre} and *Foxp3*^{Cre}*Hif2α*$^{f/f}$ mice were analyzed for expression of Foxp3, Helios and Nrp1.

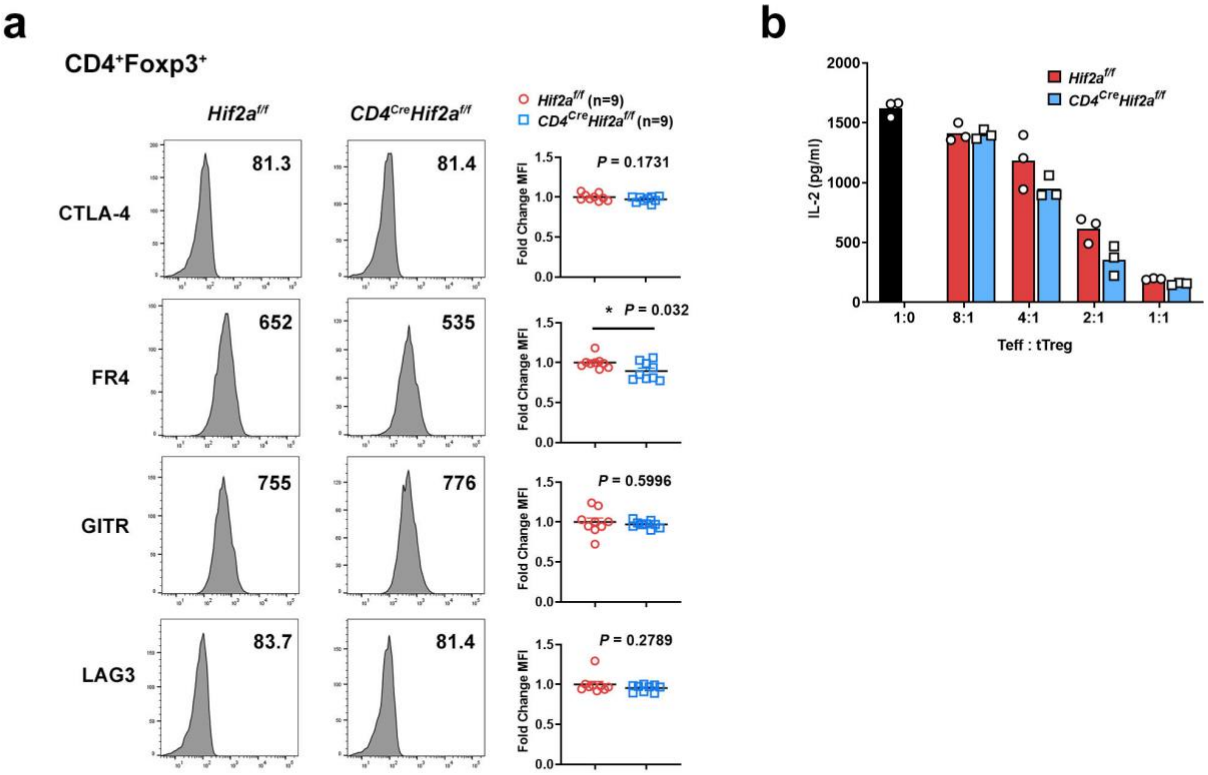

Supplementary Figure 5. Normal HIF-2α-deficient iTreg phenotypes and *in vitro* suppressive activity. (**a**) WT (*Hif2a$^{f/f}$*) and *Cd4^{Cre}Hif2a$^{f/f}$* iTregs were differentiated as in Fig. 1c. Expression of CTLA-4, GITR, FR4 and LAG3 by iTregs was determined. Numbers indicate mean fluorescence intensity (MFI). Right panel, MFI of WT (*Hif2a$^{f/f}$*) CD4$^+$Foxp3$^+$ T cells was set as 1, and ratios of MFIs of *Cd4^{Cre}Hif2a$^{f/f}$* tTregs are expressed as the mean ± SEM, n = 9 biologically independent mice. Red circle, *Hif2a$^{f/f}$*; blue square, *Cd4^{Cre}Hif2a$^{f/f}$*. **P* = 0.032 (FR4), as determined by one-way ANOVA. (**b**) Suppression by iTreg cells of splenic CD4$^+$CD25$^-$ cell activation, as measured by IL-2 production, was assessed as in Fig. 1e. Data are technical triplicates of a single experiment. The experiment was independently reproduced three times. Source data are provided as a Source Data file.

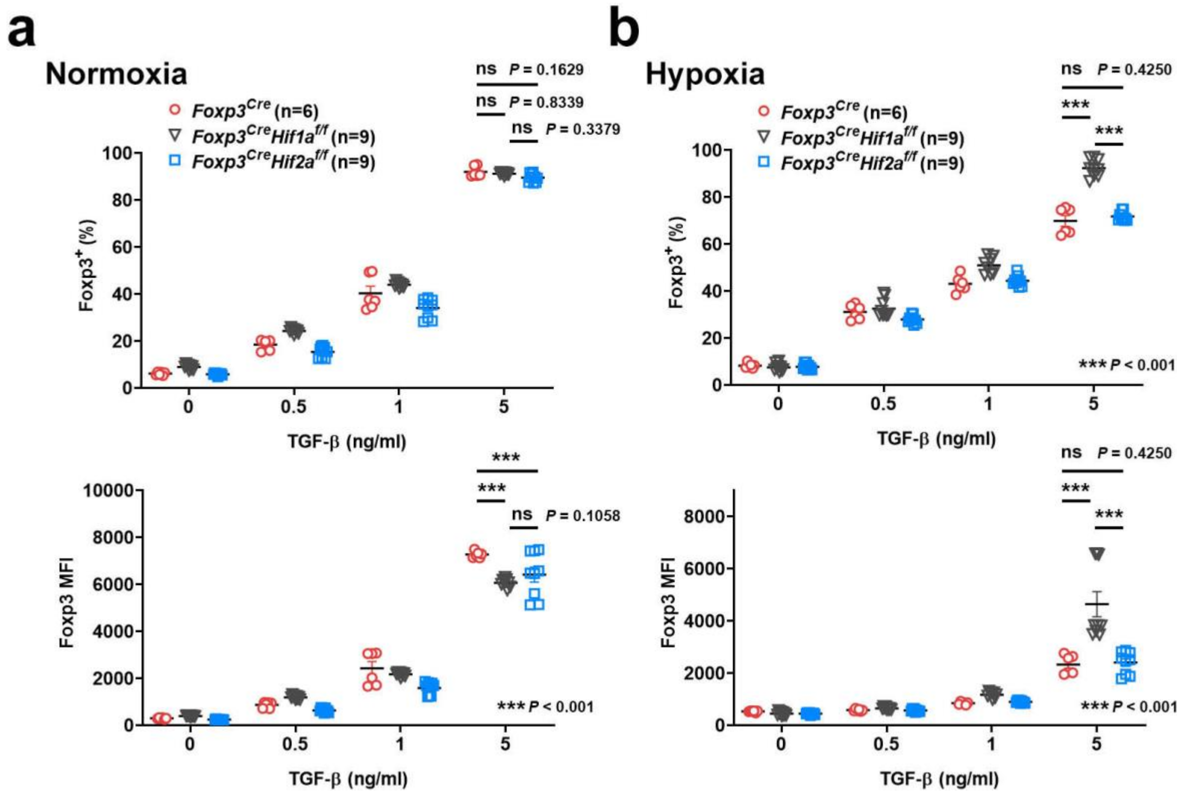

Supplementary Figure 6. iTreg differentiation under normoxia or hypoxia from *Foxp3*^{Cre}, *Foxp3*^{Cre}*Hif1a*$^{f/f}$ and *Foxp3*^{Cre}*Hif2a*$^{f/f}$ naïve T cells. **(a, b)** Naïve CD4^{+} T cells from *Foxp3*^{Cre}, *Foxp3*^{Cre}*Hif1a*$^{f/f}$ and *Foxp3*^{Cre}*Hif2a*$^{f/f}$ mice were activated with anti-mouse CD3/CD28 (2/1 µg ml^{-1}) and differentiated under different doses of TGF-β (0.5, 1, or 5 ng ml^{-1}) and mouse IL-2 (20 ng ml^{-1}) under normoxia (a) or hypoxia (1% O_2) (b). Foxp3 expression at day 3 was assessed by intracellular staining. Each data point represents iTreg derived from naïve T cells of an biologically independent mouse. Red circle, *Foxp3*^{Cre} (n = 6 mice); black inverted triangle, *Foxp3*^{Cre}*Hif1a*$^{f/f}$ (n = 9 mice); blue square, *Foxp3*^{Cre}*Hif2a*$^{f/f}$ (n = 9 mice). Foxp3 percentages and MFI were presented as the mean ± SEM. ***$P <$ 0.001 (a, b), two-way ANOVA with Tukey's multiple comparison test. ns, not significant. Source data are provided as a Source Data file.

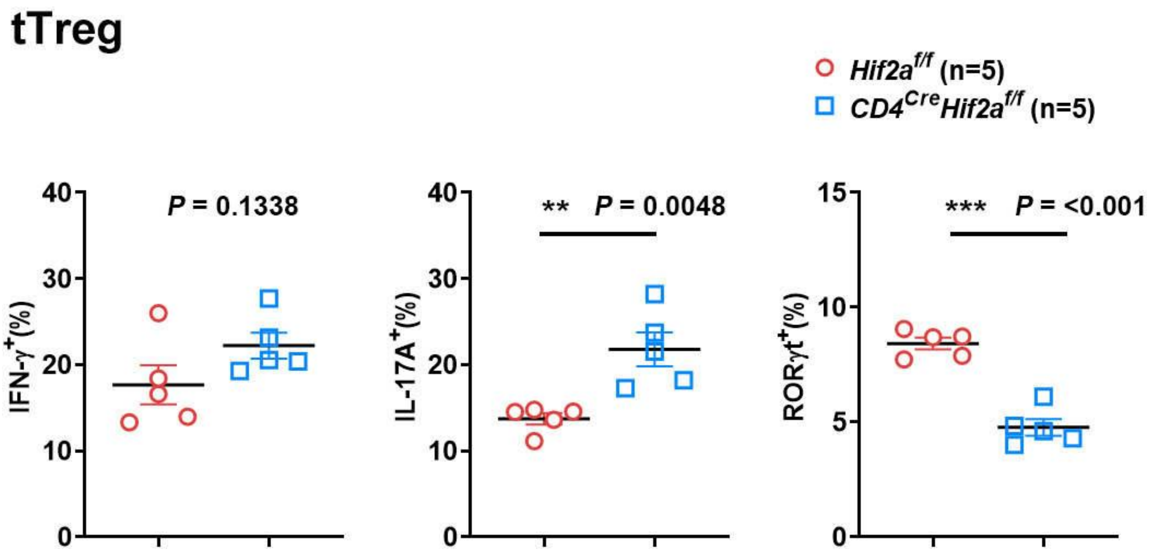

Supplementary Figure 7. RORγt expression is not increased in *Hif2a*–/– tTreg. *Hif2af/f* and *Cd4CreHif2af/f* tTregs were activated as described in Fig. 3b, and the intracellular levels of IFN-γ, IL-17 and RORγt determined by flow cytometry. Red circle, *Hif2af/f*; blue square, *Cd4CreHif2af/f*. n = 5. Data represent mean ± SEM. Statistical analyses were performed using parametric, two-tailed unpaired *t*-test. *P* = 0.1338 (IFN-γ+), ***P* = 0.0048 (IL-17A+), ****P* < 0.001 (RORγt+). Source data are provided as a Source Data file.

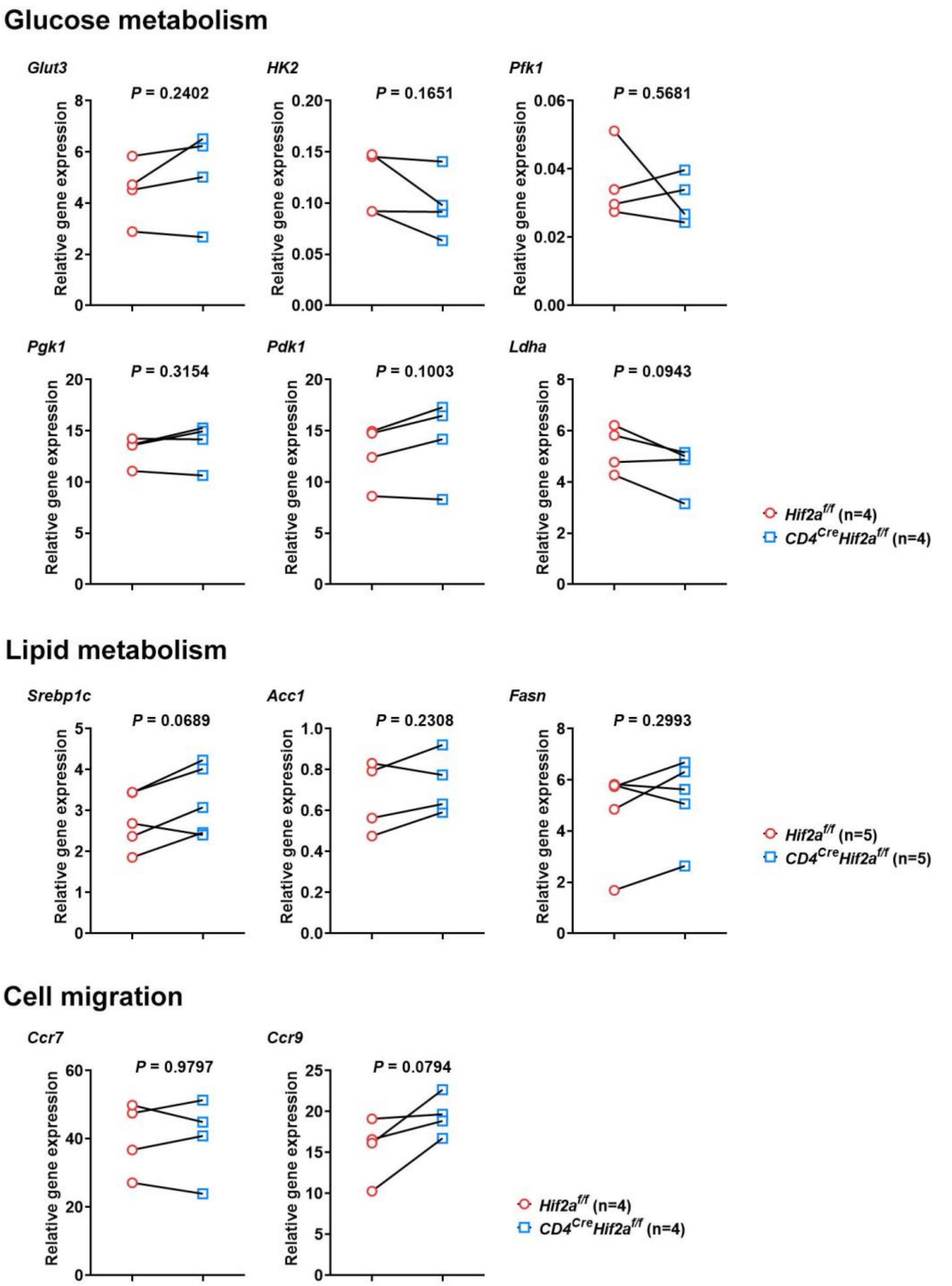

Supplementary Figure 8. Expression of several HIF-1α targets is not significantly altered in *Hif2a*$^{-/-}$ tTregs. *Hif2a*$^{f/f}$ (red circle) and *Cd4*^{Cre}*Hif2a*$^{f/f}$ (blue square) tTregs were analyzed for the expression of HIF-1α transcriptional targets involved in glucose metabolism, lipid metabolism and cell migration, and normalized against *Actin*. Each data point represented the mean average obtained from triplicate technical replicates of tTregs from an individual mouse. Red circle, *Hif2a*$^{f/f}$; blue square, *Cd4*^{Cre}*Hif2a*$^{f/f}$. n = 4 for glucose metabolism and cell migration genes, n =5 for lipid metabolism genes. The actual *P*-values are indicated (parametric, two-tailed paired *t*-test). Source data are provided as a Source Data file.

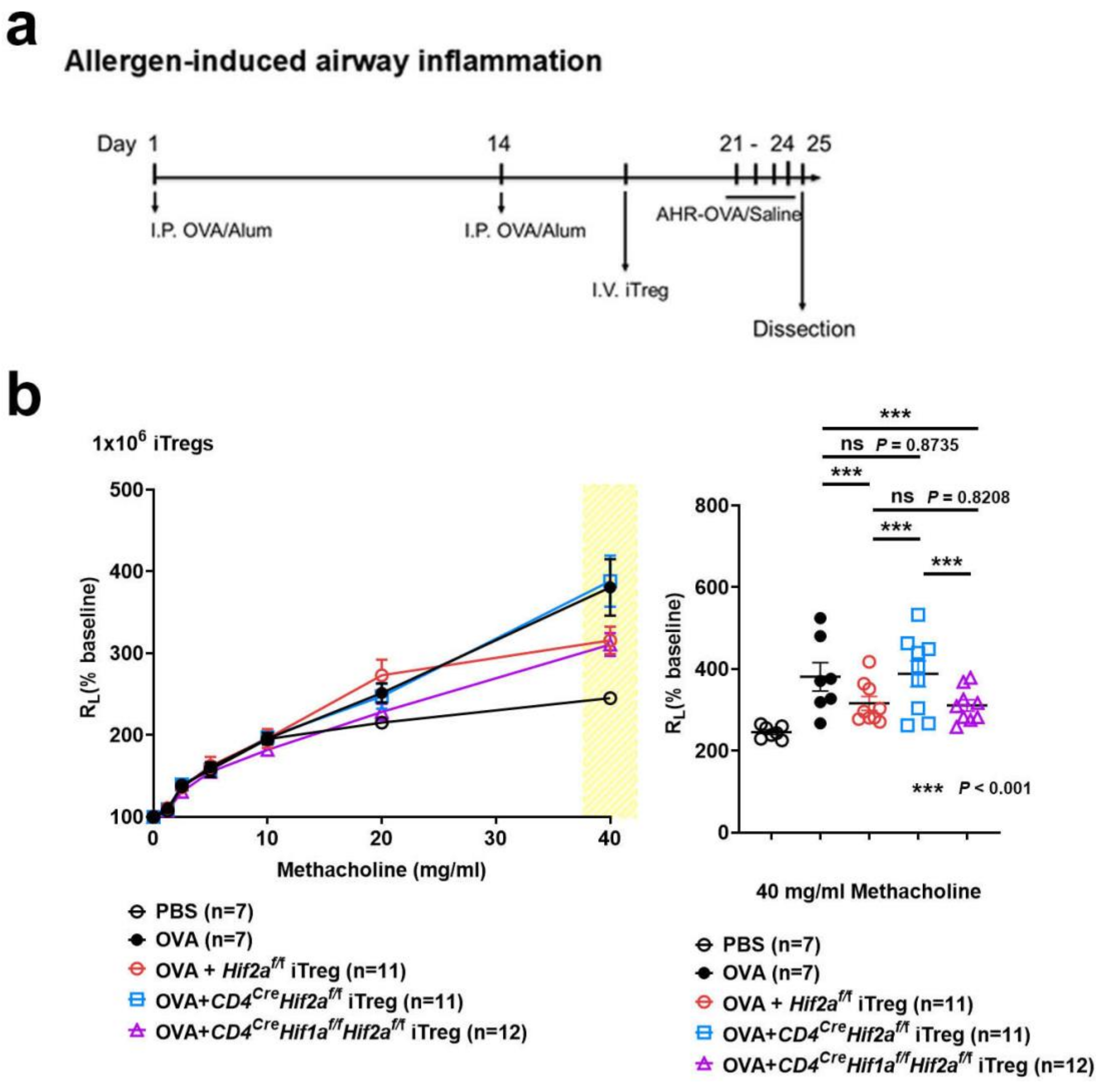

Supplementary Figure 9. Inhibition of allergenic inflammation by iTregs after allergen sensitization. **(a)** Scheme of experiment. C57BL/6 mice were sensitized with 50 mg ovalbumin (OVA) in aluminum hydroxide on days 1 and 14, followed by intravenous administration of with WT, *Hif2a*$^{-/-}$ iTregs or *Hif1a*$^{-/-}$*Hif2a*$^{-/-}$ iTregs (1×10^6) on day 18. Starting day 21, mice were challenged with an aerosol of 1% OVA in saline for 30 min for four consecutive days and then sacrificed 24 h after the last OVA challenge. **(b)** Changes in lung resistance (R_L) in response to increasing doses of methacholine were measured. Open circle, PBS (n = 7); black solid circle, OVA (n = 7); red circle, OVA + *Hif2a*$^{f/f}$ iTreg (n =11); blue square, OVA + *Cd4*^{Cre}*Hif2a*$^{f/f}$ iTreg (n = 11); purple triangle, OVA + *Cd4*^{Cre}*Hif1a*$^{f/f}$*Hif2a*$^{f/f}$ iTreg (n = 12). Right panel, lung resistance at 40 mg/ml methacholine are expressed as mean ± SEM. ***$P < 0.001$, as analyzed by two-way ANOVA with Tukey's multiple comparisons test. ns, not significant. Source data are provided as a Source Data file.

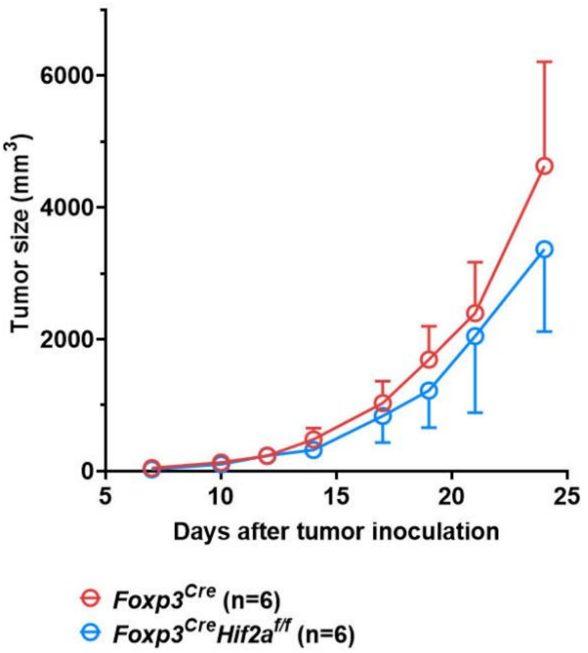

Supplementary Figure 10. HIF-2α deficiency in Tregs does not prevent growth of B16F10 melanoma. B16F10 melanoma cells (1×10^5) were subcutaneously implanted into *Foxp3*^{Cre} (red circle) and *Foxp3*^{Cre}*Hif2a*$^{f/f}$ (blue circle) mice, where *Foxp3*^{Cre} mice were littermates of *Foxp3*^{Cre}*Hif2a*$^{f/f}$ mice, and tumor growth was determined. n = 6 mice per group. Each time point represents mean ± SEM of tumor volume. Source data are provided as a Source Data file.

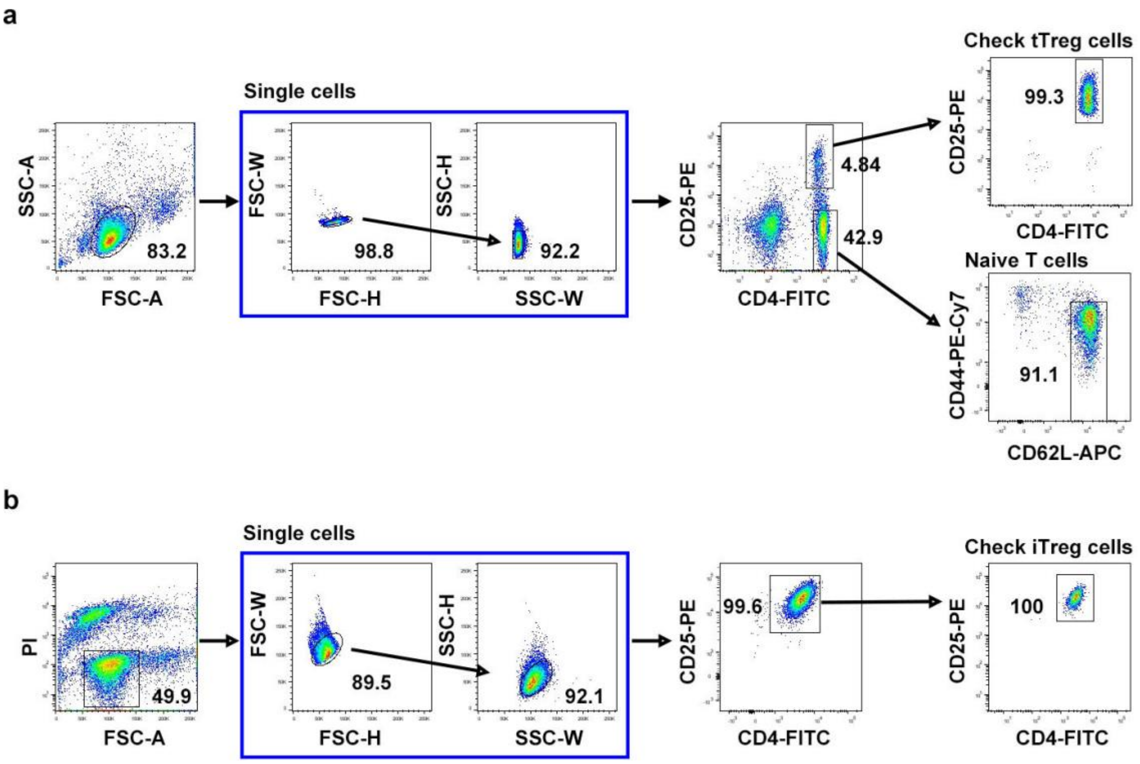

Supplementary Figure 11. FACS gating/sorting strategies of tTregs and iTregs. (a) FACS gating/sorting strategies of tTregs. Freshly isolated $CD4^+$ T cells were gated for single cells, then sorted based on the level of CD25. The sorted $CD4^+CD25^h$ population (tTreg) was checked for their purity. $CD4^+CD25^-$ population was then sorted for $CD62L^h$ as naïve $CD4^+$ T cells. **(b)** FACS gating/sorting strategies of iTregs. iTregs were differentiated from naïve $CD4^+$ T cells for 5 days as in Fig. 1c. The cell population was gated for PI^-, followed by single cells, $CD4^+CD25^h$ population was then sorted as iTregs, and the purity confirmed.

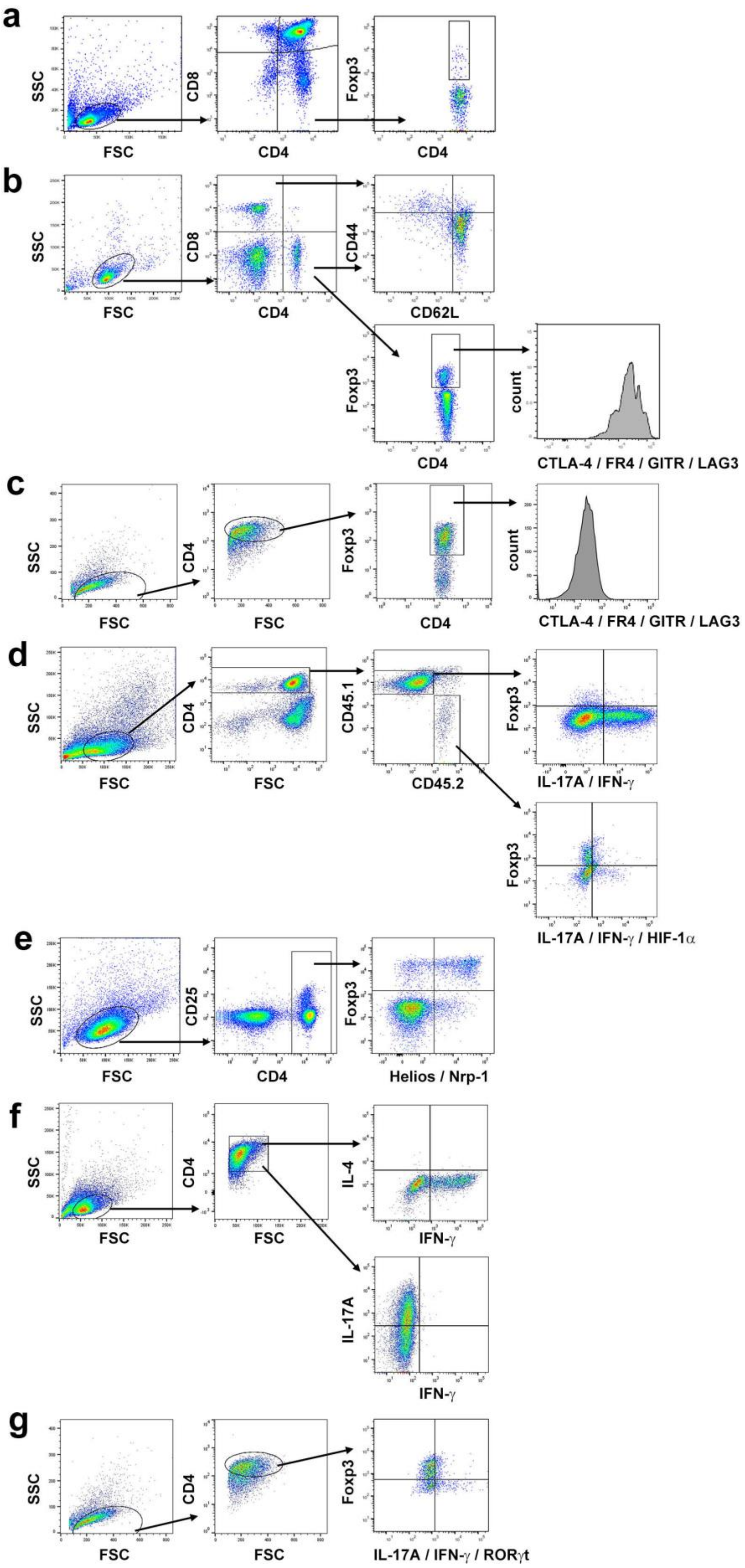
a
SSC
FSC
CD8
CD4
Foxp3
CD4
b
SSC
FSC
CD8
CD4
CD44
CD62L
Foxp3
CD4
count
CTLA-4 / FR4 / GITR / LAG3
c
SSC
FSC
CD4
FSC
Foxp3
CD4
count
CTLA-4 / FR4 / GITR / LAG3
d
SSC
FSC
CD4
FSC
CD45.1
CD45.2
Foxp3
IL-17A / IFN-γ
Foxp3
IL-17A / IFN-γ / HIF-1α
e
SSC
FSC
CD25
CD4
Foxp3
Helios / Nrp-1
f
SSC
FSC
CD4
FSC
IL-4
IFN-γ
IL-17A
IFN-γ
g
SSC
FSC
CD4
FSC
Foxp3
IL-17A / IFN-γ / RORγt

Supplementary Figure 12. FACS gating strategies. **(a)** FACS gating strategies used in Fig. 1a and Supplementary Fig. 2a, 2d, 4a. Freshly isolated thymic T cells were initially gated by forward scatter (FSC) and low side scatter (SSC) gating, followed by gating for $CD4^+$ and $CD8^+$ single positive T cells. $CD4^+$ T cells were gated for $Foxp3^+$ population. **(b)** FACS gating strategies used in Fig. 1a, 1b, and Supplementary Fig. 2b-2e, 4b-4c. Freshly isolated lymphocytes from spleen or lymph node were initially gated with FSC and low SSC. $CD4^+$ and $CD8^+$ T cells were gated, followed by gating for $CD62L^+CD44^-$ (naïve) and $CD62L^-CD44^+$/$CD62L^+CD44^+$ (effector/memory). $CD4^+$ T cells were also gated for $Foxp3^+$ population to assess the levels of CTLA4, FR4, GITR, and LAG-3. **(c)** FACS gating strategies used in Fig. 1c, 1d, and Supplementary Fig. 5a, 6a, 6b. Differentiated iTregs were gated for $CD4^+$ followed by gating for $Foxp3^+$ population to determine the expression of CTLA4, FR4, GITR, and LAG-3. **(d)** FACS gating strategies used in Fig. 2e-2g, 4g and 5a. Lymph node cells of the recipient $CD45.1^+$ mice were initially gated by FSC and low SSC. $CD4^+$ T cells were gated for $CD45.1^+$ and $CD45.2^+$ populations. $CD45.1^+$ and $CD45.2^+$ T cells were then gated for intracellular expression of IL-17A, IFN-γ, HIF-1α and Foxp3. **(e)** FACS gating strategies used in supplementary 2f and 4d. Freshly isolated lymphocytes from lymph nodes were first gated by FSC and SSC. The $CD4^+$ T cells were gated for subpopulations of $Foxp3^+$ versus $Nrp\text{-}1^+$ or $Helios^+$. **(f)** FACS gating strategies used in Fig. 3f. Different Th cells differentiated from naïve T cells were first gated with FSC and SSC. The $CD4^+$ T cells were gated for intracellular production of IFN-γ, IL-4 and IL-17A. **(g)** FACS gating strategies used in Supplementary Fig. 7. $CD4^+Foxp3^+$ tTregs primed for different Th cells were initially gated by FSC and SSC. $CD4^+$ T cells were then gated for intracellular expression of Foxp3, IL-17A, IFN-γ, and RORγt.

Fig. 5d

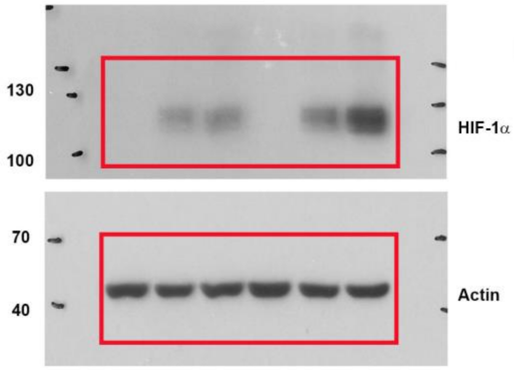

Fig. 5f

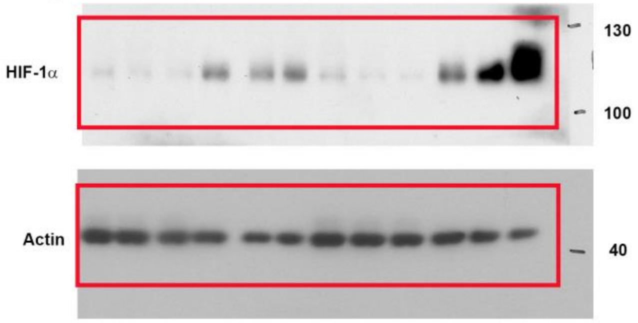

Fig. 5e

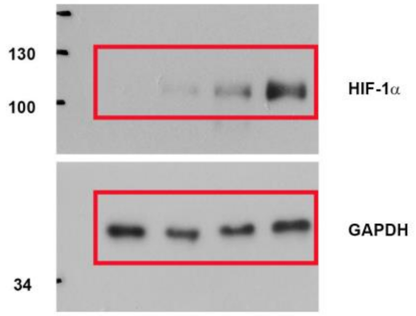

Fig. 5g

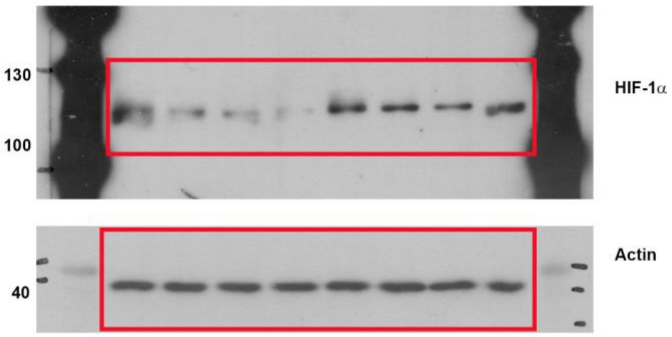

Fig. 5h

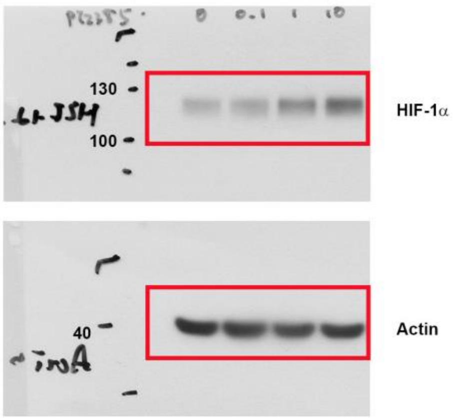

Fig. 5i

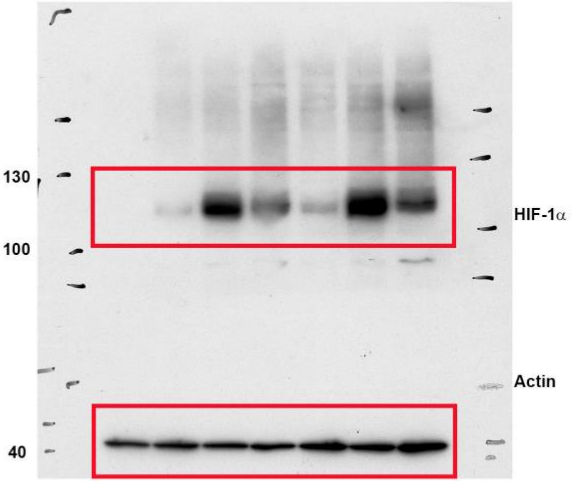

Supplementary Figure 13. Uncropped images of the original scans of immunoblots. Uncropped, full-size scans of immunoblots shown in Fig. 5d, 5e, 5f, 5g, 5h, 5i.

Supplementary Table 1. Primer sequences for real-time quanti

Target gene	Forward	Reverse
Hif1a	TGAGCTTGCTCATCAGTTGC	CCATCTGTGCCTTCATCTCA
Hif2a	TGAGTTGGCTCATGAGTTGC	TTGCTGATGTTTTCCGACAG
Glut1	GGATCTCTCTGGAGCACAGG	TCCTCCTGGACTTCACTGCT
Glut3	CTCTTCAGGTCACCCAACTACGT	CCGCGTCCTTGAAGATTCC
Hk2	TGATCGCCTGCTTATTCACGG	AACCGCCTAGAAATCTCCAGA
Pfk1	GGTGTACAAGCTTCTAGCTC	CAAGTTTAGAGCCACCTTGG
Pgk1	CTGTGGTACTGAGAGCAGCAAGA	CAGGACCATTCCAAACAATCTG
Pdk1	GCAGCAGAGAGTAAACTGTTTG	TGGTCACCTGACCTCTCG
Ldha	TGGCAGACTTGGCTGACAG	ACCTTCACAACATCCGAGATTC
Srebp1c	GAACAGACACTGGCCGAGAT	GAGGCCAGAGAAGCAGAAGAG
Acc1	ACAGTGGAGCTAGAATTGGAC	ACTTCCCGACCAAGGACTTTG
Fasn	AGCGGCCATTTCCATTGCCC	CCATGCCCAGAGGGTGGTTG
Ccr4	GGAAGGTATCAAGGCATTTGGG	GTACACGTCCGTCATGGACTT
Ccr7	ATGCTGGCTATGAGTTTC	GCTGCTATTGGTGATGTT
Ccr9	CACCATGATGCCCACAGAAC	GATGAGAAGCACACAGCTGTAG
Actin	GGCTACAGCTTCACCACCAC	ATGCCACAGGATTCCATACC
