# Supplementary material for: HIF-2α is indispensable for regulatory T cell function
Source: Nat Commun. 2020 Oct 6;11:5005. doi: 10.1038/s41467-020-18731-y (PMC7538433; doi:10.1038/s41467-020-18731-y)
Supplement: Supplementary file 3 — Reporting Summary [file 41467_2020_18731_MOESM3_ESM.pdf]

nature research

Corresponding author(s): Ming-Zong Lai NCOMMS-19-27974A

Last updated by author(s): 2020/08/21

Reporting Summary

Nature Research wishes to improve the reproducibility of the work that we publish. This form provides structure for consistency and transparency in reporting. For further information on Nature Research policies, see Authors & Referees and the Editorial Policy Checklist .

Statistics

For all statistical analyses, confirm that the following items are present in the figure legend, table legend, main text, or Methods section.

n/a	Confirmed	
☐	☒	The exact sample size (*n*) for each experimental group/condition, given as a discrete number and unit of measurement
☐	☒	A statement on whether measurements were taken from distinct samples or whether the same sample was measured repeatedly
☐	☒	The statistical test(s) used AND whether they are one- or two-sided<br>*Only common tests should be described solely by name; describe more complex techniques in the Methods section.*
☒	☐	A description of all covariates tested
☐	☒	A description of any assumptions or corrections, such as tests of normality and adjustment for multiple comparisons
☐	☒	A full description of the statistical parameters including central tendency (e.g. means) or other basic estimates (e.g. regression coefficient) AND variation (e.g. standard deviation) or associated estimates of uncertainty (e.g. confidence intervals)
☐	☒	For null hypothesis testing, the test statistic (e.g. *F*, *t*, *r*) with confidence intervals, effect sizes, degrees of freedom and *P* value noted<br>*Give P values as exact values whenever suitable.*
☒	☐	For Bayesian analysis, information on the choice of priors and Markov chain Monte Carlo settings
☒	☐	For hierarchical and complex designs, identification of the appropriate level for tests and full reporting of outcomes
☒	☐	Estimates of effect sizes (e.g. Cohen's *d*, Pearson's *r*), indicating how they were calculated

Our web collection on statistics for biologists contains articles on many of the points above.

Software and code

Policy information about availability of computer code

Data collection	Flow cytometry data were collected on FACSAria II SORP or LSRII-18P with FACSDiva software version 8.0.2 (BD).<br>qRT-PCR data were collected with LightCycler 480 Real-Time PCR System software version 1.5.1.62.
Data analysis	Flow cytometry data were analyzed on FlowJo version 10 (BD) and statistical analysis performed on Prism version 5 (GraphPad).<br>Microsoft Office Excel version 2016 were used for data analysis.

For manuscripts utilizing custom algorithms or software that are central to the research but not yet described in published literature, software must be made available to editors/reviewers. We strongly encourage code deposition in a community repository (e.g. GitHub). See the Nature Research guidelines for submitting code & software for further information.

Data

Policy information about availability of data

All manuscripts must include a data availability statement. This statement should provide the following information, where applicable:

- Accession codes, unique identifiers, or web links for publicly available datasets
- A list of figures that have associated raw data
- A description of any restrictions on data availability

The source data underlying figures and supplementary figures are provided as a Source Data file.

Field-specific reporting

Please select the one below that is the best fit for your research. If you are not sure, read the appropriate sections before making your selection.

Life sciences study design

All studies must disclose on these points even when the disclosure is negative.

Sample size	No sample size calculation was performed. Based on our experience in Treg study, the original plan was to get at least 4 mice each group or 4 biologically independent samples each in vitro experiments. That was the way conducted in most of experiments in this study. However, a few experiments were limited by the number of the specific knockout mice and their normal breeding control available. These few experiments were settled for 3 mice or 2-3 biologically independent samples if statistic significance was obtained or if the experiment was confirmatory in nature (of previous reports). The value of N was indicated in the figure and legend.
Data exclusions	No data was excluded in this study.
Replication	All experiments presented in this study were performed using at least 2-3 biological replicates. All experiments have been reproducibly repeated independently. All replications gave similar results.
Randomization	Mice were randomized after tumor inoculation to obtain homogeneous groups. Recipient mice matched by age and sex were randomized for colitis and airway inflammation experiments. For in vitro experiments, Treg from individual mouse of the same genetic modification was randomized for treatments (e.g. Th17 polarization, PT2385) or analysis.
Blinding	Blinding was implemented in airway inflammation experiments and histology (colitis) in this study. Several experiments(e.g. in vitro Treg suppression) were independently performed and confirmed by two different persons. Blinding was not performed in other experiments. For some experiments (e.g. colitis), the readout (body weight loss) is clearcut. For other experiments, because of the consistent expertise required to carry out different parts of the experiments, the experiment was conducted by the same person (first author).

Reporting for specific materials, systems and methods

We require information from authors about some types of materials, experimental systems and methods used in many studies. Here, indicate whether each material, system or method listed is relevant to your study. If you are not sure if a list item applies to your research, read the appropriate section before selecting a response.

Materials & experimental systems

n/a	Involved in the study	
☐	☒	Antibodies
☐	☒	Eukaryotic cell lines
☒	☐	Palaeontology
☐	☒	Animals and other organisms
☒	☐	Human research participants
☒	☐	Clinical data

Methods

n/a	Involved in the study	
☒	☐	ChIP-seq
☐	☒	Flow cytometry
☒	☐	MRI-based neuroimaging

Antibodies

Antibodies used

-> For flow cytometry:
Anti-IL-4-PE (11B11, 1:250), anti-CD8-APC (53-6.7, 1:500), anti-CD62L-APC (MEL-14, 1:500), anti-CD25-FITC (PC61, 1:1000), anti-CD25-PE (PC61, 1:1000), anti-GITR-PE (YGITR 765, 1:250), anti-IFN-γ-APC (XMG1.2, 1:250), anti-IL-17-FITC (TC11-18H10.1, 1:250), anti-mouse/human Helios-PE (22F6, 1:250), and anti-mouse CD304-Brilliant Violet 421 (Neutropilin-1, 3E12, 1:500) were purchased from BioLegend (San Diego, CA). Anti-CD4-FITC (GK1.5 and RM4-5, 1:500), anti-CD44-PE-Cy7 (IM7, 1:1000), anti-CTLA-4-PE (clone UC10-4B9, 1:250), anti-FR4-PE (clone eBio12A5, 1:250), anti-Foxp3-Pacific blue or APC (FJK-16S, 1:200), anti-LAG-3-PE (eBioC9B7W, 1:250), anti-CD45.1-PE (A20, 1:500), and anti-CD45.2-PE-Cy7 (104, 1:500) were purchased from Thermo Fisher (Madison, WI).

-> For Western blot:
Antibodies against GAPDH (6C5, 1:6000) was obtained from Santa Cruz Biotechnology (Santa Cruz, CA). Antibodies against HIF-1alpha(10006421, 1:2000) were purchased from Cayman Chemical (Ann Arbor, MI). Antibodies against actin (MAB1501, C4, 1:4000) were purchased from Merck Millipore (Billerica, MA).

-> For functional assay and cell purification:
Anti-CD3 (2C11) and anti-CD28 (37.51) were purchased from BioXCell (West Lebanon, NH). Anti-IL-2 (JES6-1A12), anti-IL-4 (11B11), anti-IL-12 (C17.8), anti-IFN-γ (R4-6A2) were purchased from BioLegend. Anti-mouse CD4 (RL172.4) was purified in our laboratory.

Validation

Flow cytometry antibodies used in this study from BioLegend were validated by endogenous positive and negative expressing cells, knockdown/knockout cells and isotype controls. Functional assay antibodies from BioLegend were validated by biological testing and ELISA capture. Flow cytometry antibodies from Thermo Fisher were validated by target specificity verification and flow cytometry application validation. Anti-actin from Merck Millipore was verified by Western blot detection of endogenous actin in HEK293 cell lysate. Anti-HIF-1alpha from Cayman was verified by Western blot detection of HIF-1alpha in lysate from

PHD inhibitor-treated COS cell. Anti-GAPDH from Santa Cruz was verified by Western blot detection of GAPDH in GAPDH-transfected 293T and HepG2 as well as by literature. Anti-CD3, anti-CD28 and anti-CD4 were verified by functional assay in our laboratory.

Eukaryotic cell lines

Policy information about cell lines

Cell line source(s)	B16F10 melanoma cells were obtained from ATCC (CRL-6475, Manassas, VA). MC38 colon adenocarcinoma cells were purchased from Kerafast (CVCL_B288, Boston, MA). MC38 and B16F10 cells were cultured in DMEM (Life Technologies, Carlsbad, CA) supplemented with 10% FCS (Life Technologies), 1% Penicillin-Streptomycin, 1% L-Glutamine, and 1% HEPES.
Authentication	Both cell lines were unauthenticated.
Mycoplasma contamination	Cell lines were not tested for mycoplasma contamination, but were treated with Mycoplasma Removal agents (MP Biomedicals) before experiments.
Commonly misidentified lines (See ICLAC register)	No commonly misidentified cell lines were used in the study.

Animals and other organisms

Policy information about studies involving animals; ARRIVE guidelines recommended for reporting animal research

Laboratory animals	Cd4-Cre mice were obtained from Taconic Farms. Rag1-/- mice, Hif1atm3Rsjo/J (Hif1af/f) mice, Epas1tm1Mcs/J (Hif2a f/f) mice, and NOD/ShiLt-Tg(Foxp3-EGFP/cre)1cJbs/J mice (also known as NOD.Foxp3-Cre) were obtained from Jackson Laboratories. NOD.Foxp3-Cre mice were back-crossed to C57BL/6 mice for 12 generations (Foxp3-Cre) 44. Cd4-Cre mice were bred with Hif1a f/f or Hif2a f/f for T cell-specific deletion of Hif1a or Hif2a, respectively. Foxp3-Cre mice were crossed with Hif1af/f or Hif2af/f to produce mice with Treg-specific deletion of Hif1a or Hif2a. In vivo experiments were conducted on sex-matched male and female mice. Both male and female mice were used for in vitro and in vivo experiments. All mice used in our study were 8-10 weeks old.<br>Mouse housing condition: ambient temperature at 21 °C, humidity of 55%, dark/light cycle of 10 h/14 h, and air exchange rate of 12-15 times per hour.
Wild animals	No wild animals were used in the study.
Field-collected samples	No field collected samples were used in the study.
Ethics oversight	All mouse experimental protocols were approved by the Institutional Animal Care and Utilization Committee of Academia Sinica.

Note that full information on the approval of the study protocol must also be provided in the manuscript.

Flow Cytometry

Plots

Confirm that:

- [x] The axis labels state the marker and fluorochrome used (e.g. CD4-FITC).
- [x] The axis scales are clearly visible. Include numbers along axes only for bottom left plot of group (a 'group' is an analysis of identical markers).
- [x] All plots are contour plots with outliers or pseudocolor plots.
- [x] A numerical value for number of cells or percentage (with statistics) is provided.

Methodology

Sample preparation	-> For cell sorting:<br>Single-cell suspensions were prepared from spleen and lymph nodes by crushing on 100m cell strainers. Cells were run through cell strainer and red blood cells were lysed in Red blood lysis buffer for 5 min. Total T cells were isolated by using anti-mouse Ig panning. Specific T cell populations were then sorted on FACSAria II SORP.<br><br>-> For cell analysis:<br>Cell surface and intracellular staining were performed on single-cell suspensions. For intracellular staining, cell were fixed and permeabilized using the Foxp3/Transcriptional Factor Staining Buffer Set (Thermo Fisher).
Instrument	FACSAria II SORP was used for sorting; LSRII-18P flow cytometry system was used for cell analysis.
Software	FACSDiva software (BD) and FlowJo v10 (BD).

Cell population abundance	Naïve T (CD4+CD25-CD62L+CD44-), nTreg (CD4+CD25+), effector T (CD4+CD25-), and differentiated iTreg (CD4+CD25+) cells sorted by FACSAria II SORP were typically >99%.
Gating strategy	Lymphocytes were first gated with FSC and low SSC. CD4+ T cells were gated, followed by gating for Foxp3+ population and/or intracellular production of IL-17A and IFN-gamma. For monitor of adoptively transferred T cells, CD4+ T cells were gated for CD45.1+ and CD45.2+ population before further analysis. For additional details, refer to Supplementary Figure 11 and 12.

☒ Tick this box to confirm that a figure exemplifying the gating strategy is provided in the Supplementary Information.
